# Supplementary figures and images for: Structural basis of G protein–Coupled receptor CMKLR1 activation and signaling induced by a chemerin-derived agonist
Source: PLoS Biol. 2023 Dec 6;21(12):e3002188. doi: 10.1371/journal.pbio.3002188 (PMC10699647; doi:10.1371/journal.pbio.3002188)

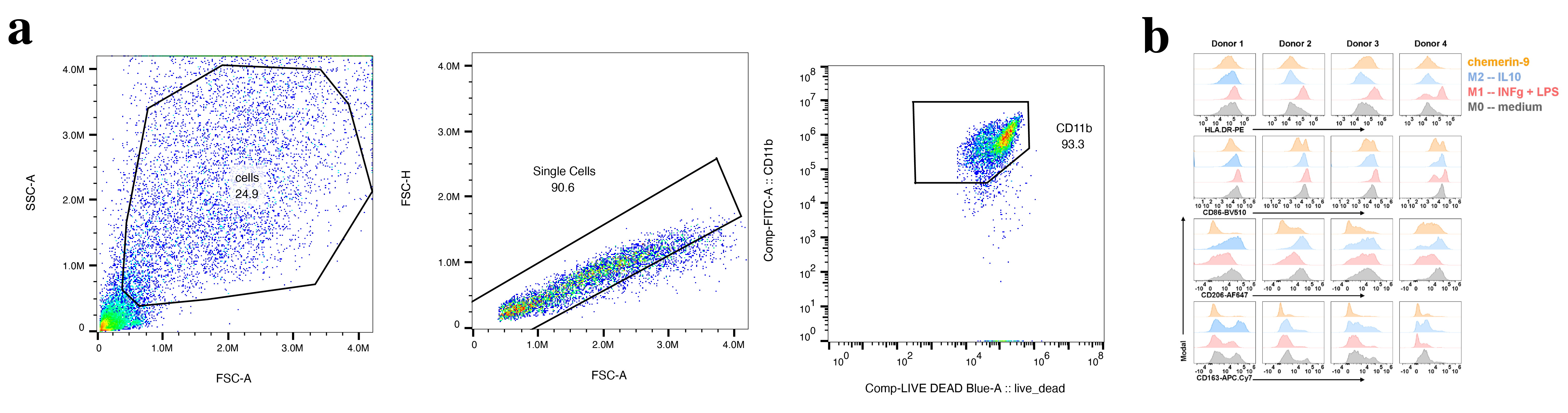

Supplement: S1 Fig — (a) Successive plots and gates that were used in the flow cytometry experiments. (b) Source flow cytometry histogram of HLA-DR, CD86, CD206, and CD163 in primary human macrophages from 4 donors. The macrophages were gated on live CD11b population associated with the interleaved scatter plot shown in the main Fig 1A. The underlying data for S1 Fig can be found in S1 Data. (JPG) [file pbio.3002188.s001.jpg]

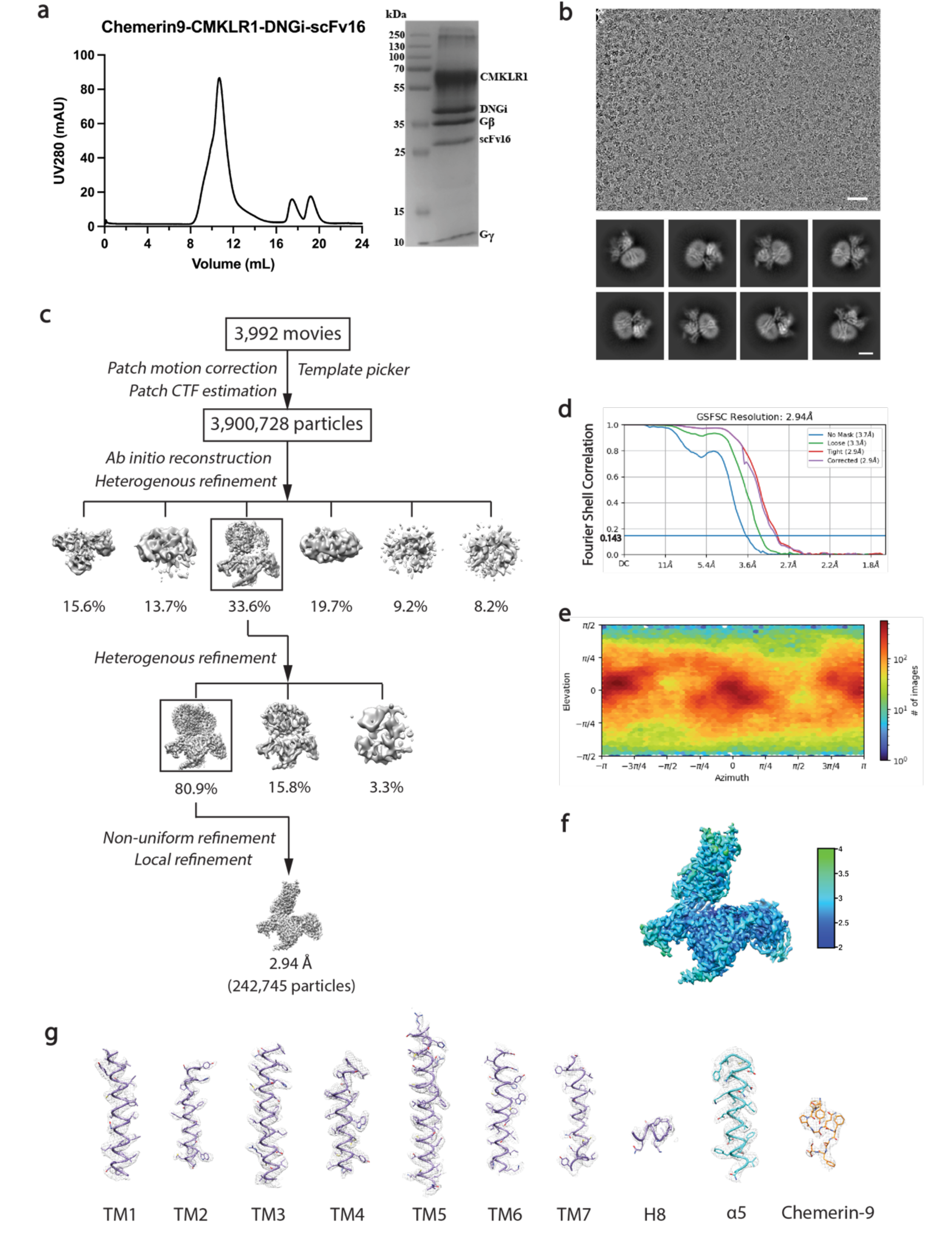

Supplement: S2 Fig — (a) Size exclusion chromatography profile and SDS-PAGE analysis of the purified Chemerin9-CMKLR1-Gi complex. (b) Representative cryo-EM micrograph (scale bar: 50 nm) and 2D class averages (scale bar: 5 nm). (c) cryo-EM image processing workflow for the Chemerin9-CMKLR1-Gi complex. (d) Gold-standard FSC curve showing an overall resolution is 2.94 Å at FSC = 0.143. (e) Angular distribution of the particles used in the final reconstruction. (f) Density map according to local resolution estimation. (g) cryo-EM density maps and models of the 7 transmembrane helices (TM1-7), Helix 8 (H8), α5 helix of Gαi, and the ligand of Chemerin9-bound CMKLR1-Gi complex are shown. The EM density is shown at 0.148 threshold. CMKLR1, chemokine-like receptor 1; cryo-EM, cryo-electron microscopy; EM, electron microscopy; FSC, Fourier shell correlation. (TIF) [file pbio.3002188.s002.tif]

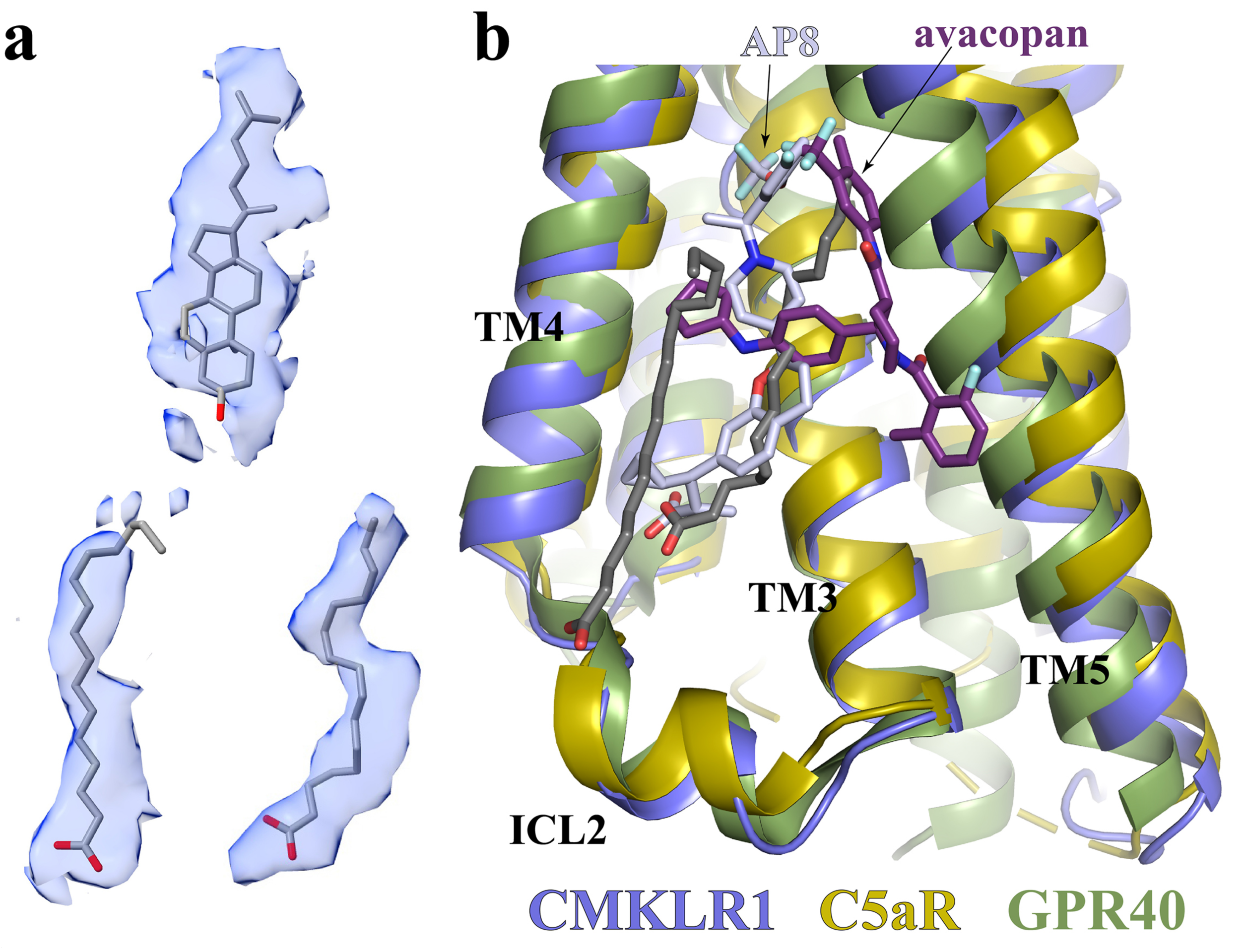

Supplement: S3 Fig — (a) cryo-EM density of 1 cholesterol molecule (upper, level = 0.1) and 2 palmitic acid molecules (lower, level = 1.5). (b) Superimposition of CMKLR1, C5aR with avacopan (PDB ID 6C1R), and GPR40 with AP8 (5TZY). The palmitic acid molecules are shown as grey sticks. Avacopan is a NAM of C5aR shown as purple sticks. AP8 is a PAM of GPR40 shown as light blue sticks. CMKLR1, chemokine-like receptor 1; cryo-EM, cryo-electron microscopy; NAM, negative allosteric modulator; PAM, positive allosteric modulator; 7-TMs, 7 transmembrane helices. (TIF) [file pbio.3002188.s003.tif]

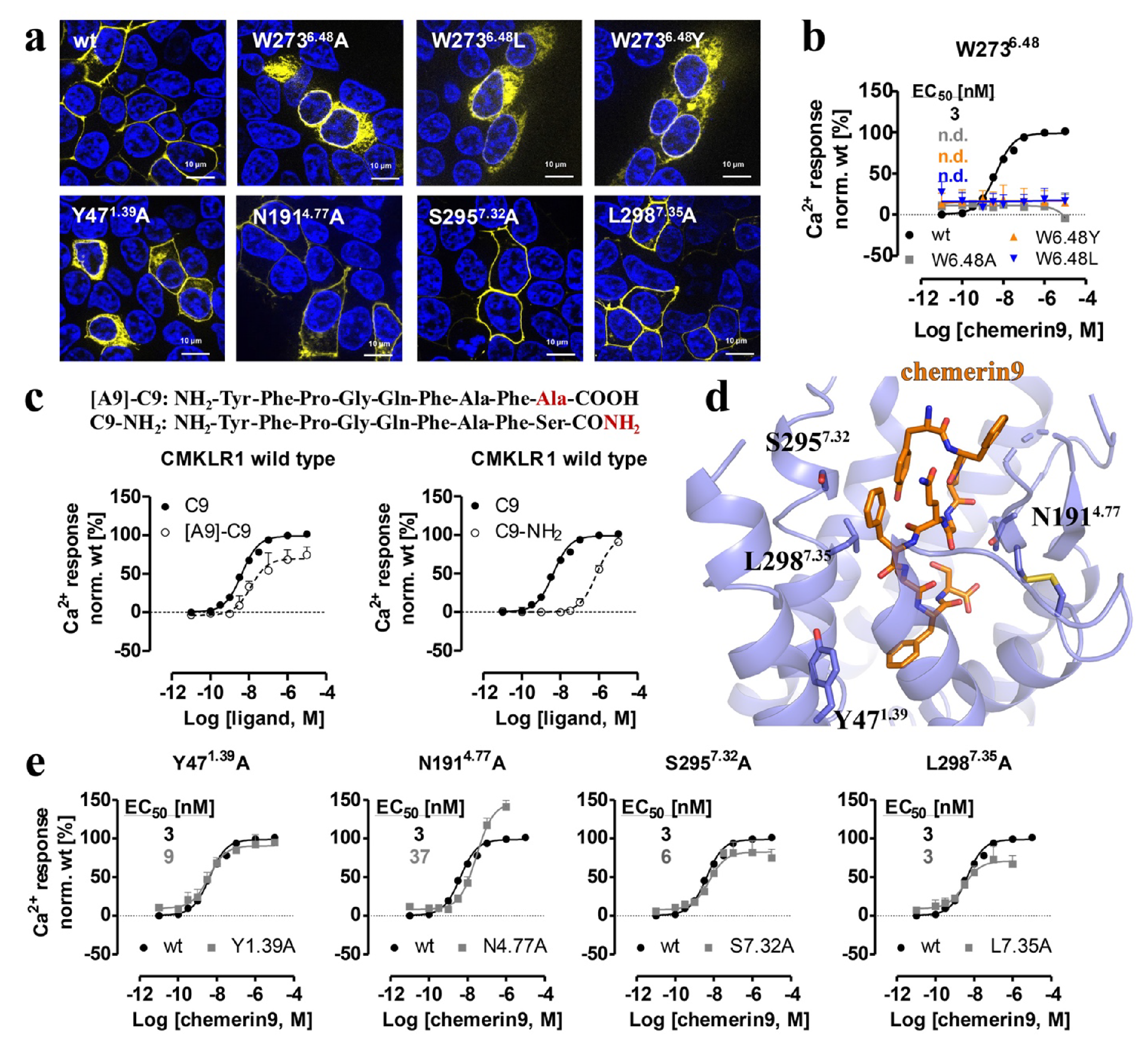

Supplement: S4 Fig — (a) Localization of CMKLR1 variants in HEK293 cells measured by eYFP autofluorescence. Nuclei were labeled with Hoechst 33342. Scale bar: 10 μm. wt CMKLR1 is expressed only in the cell membrane as well as N1914.77, S2957.32, and L2987.35. Y471.39 is mainly expressed in the membrane but partially within the cell, whereby all 3 W2736.48 variants are located exclusively in the cytosol. (b) G protein signaling of wt CMKLR1 and W2736.48 variants stimulated by C9. The receptor variants showed no Ca2+ release probably because of the receptor localization. (c) G protein signaling of wt CMKLR1 simulated by C9, [A9]-C9, and C9-NH2. The amidated C-terminus has an increased influence compared to the Ala change at position 9 in C9. (d) Positions of Y471.39, N1914.77, S2957.32, and L2987.35 of CMKLR1 and (e) signaling of their Ala variants stimulated by C9. All signaling assays were performed by measuring Ca2+ influx. The assays were executed at least 3 times in triplicates. Results are represented as means ± SEM. The underlying data for S4A, S4B and S4E Fig can be found in S5 Data. CMKLR1, chemokine-like receptor 1; C9, chemerin9; eYFP, enhanced yellow fluorescent protein; wt, wild-type. (TIF) [file pbio.3002188.s004.tif]

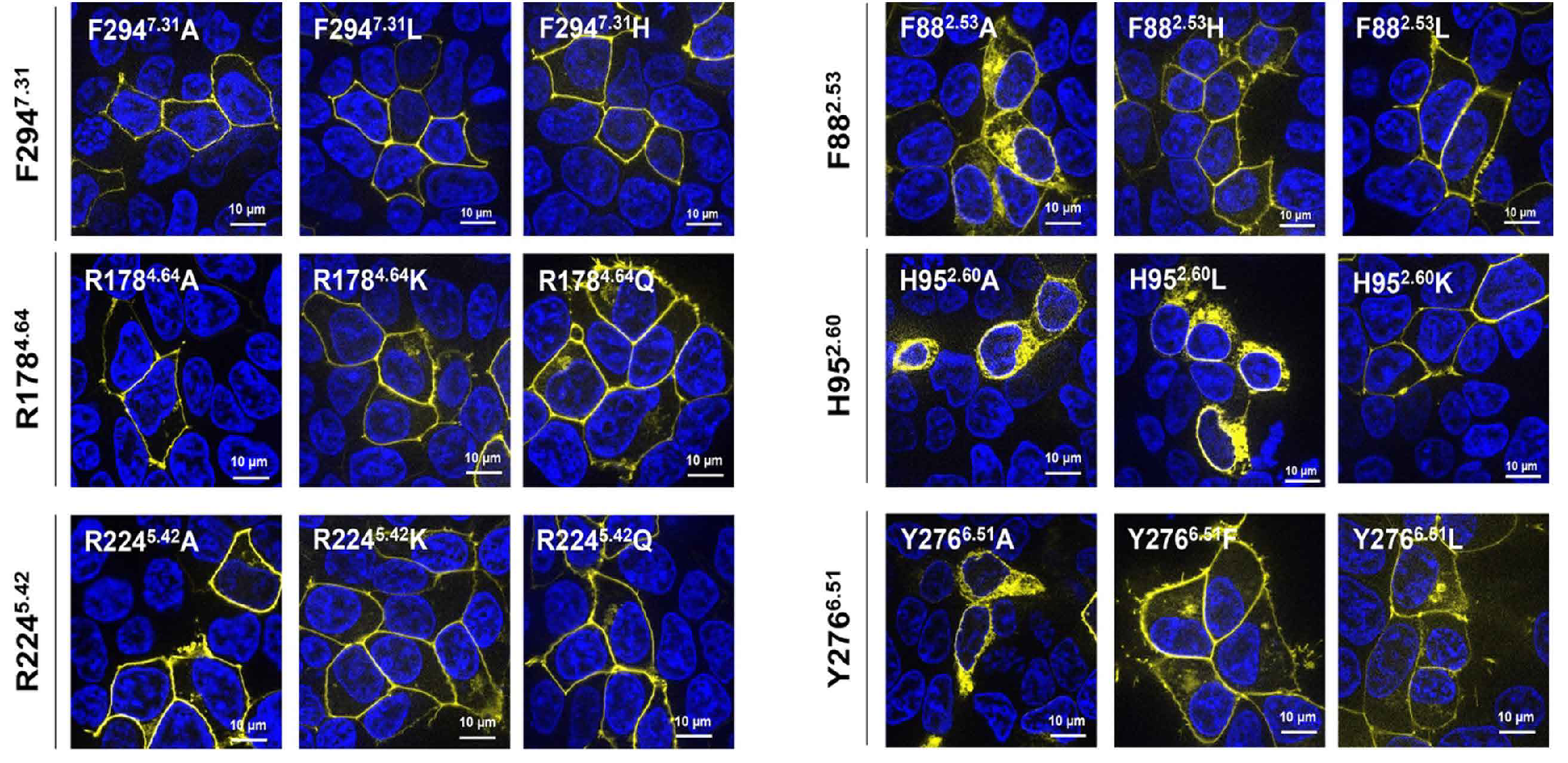

Supplement: S5 Fig — HEK293 cells were transiently transfected with 1,000 ng of receptor variant fused with eYFP with Lipofectamine 2000. Receptor expression was determined by eYFP autofluorescence. Cell nuclei were stained with Hoechst33324 (blue). Scale bar 10 μm. Most receptor variants are expressed in the membrane, but Y2766.51A, H952.60A, and H952.60L are located within the cell, whereby the F882.53A variant is only partially expressed in the membrane. (TIF) [file pbio.3002188.s005.tif]

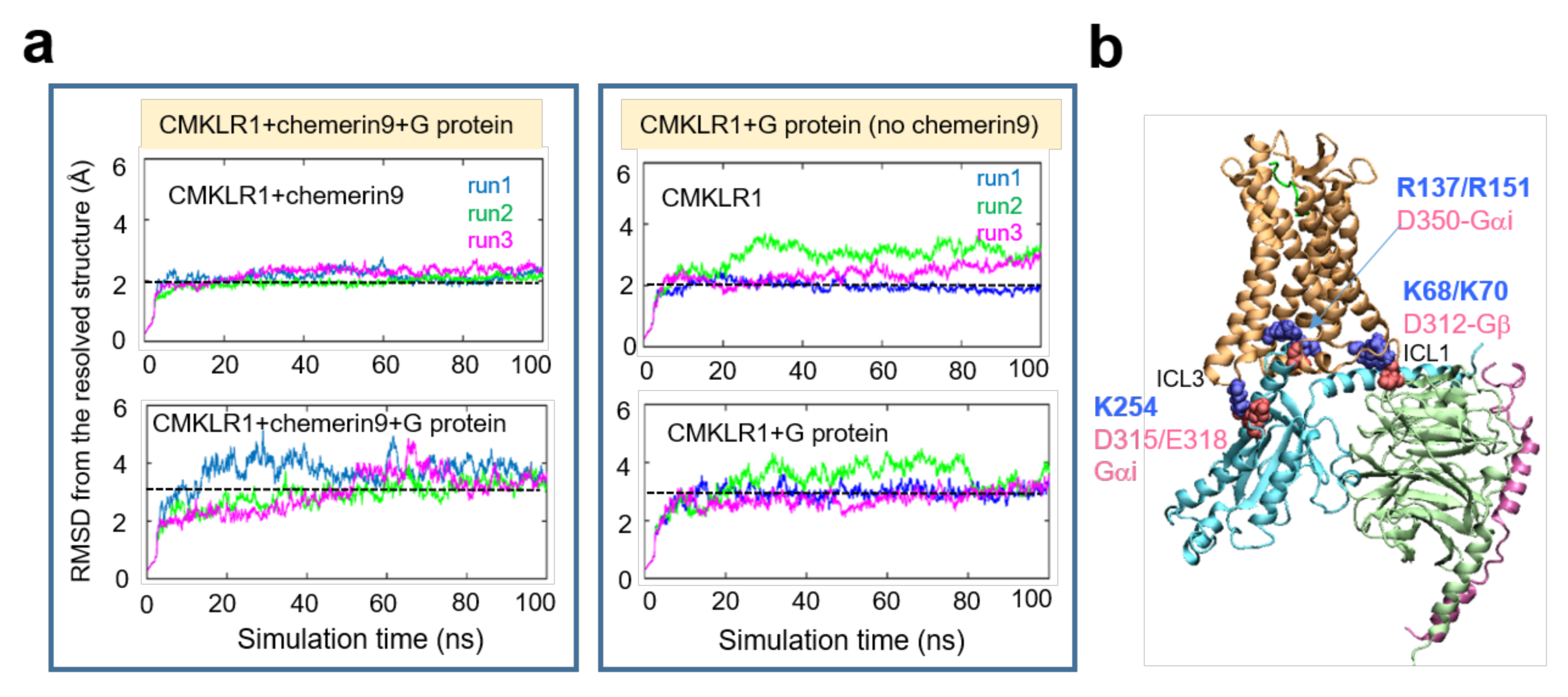

Supplement: S6 Fig — (a) RMSD of the receptor alone and the CMKLR1-Gi complex in the simulations of the CMKLR1-Gi complex with (left) and without (right) chemerin9. CMKLR1 deviated from that in the cryo-EM structure within 2.0 ± 0.5 Å, and the entire protein complex (CMKLR1 + chemerin9 + G protein) deviated from that in the cryo-EM structure within 3.5 ± 1.0 Å. In the absence of chemerin9, CMKLR1 showed more fluctuations. The underlying data for S6A Fig can be found in S6 Data. (b) Interfacial salt bridges intermittently formed between CMKLR1 and the Gi complex during the simulations. CMKLR1, chemokine-like receptor 1; cryo-EM, cryo-electron microscopy; MD, molecular dynamics; RMSD, root-mean-square deviation. (TIF) [file pbio.3002188.s006.tif]

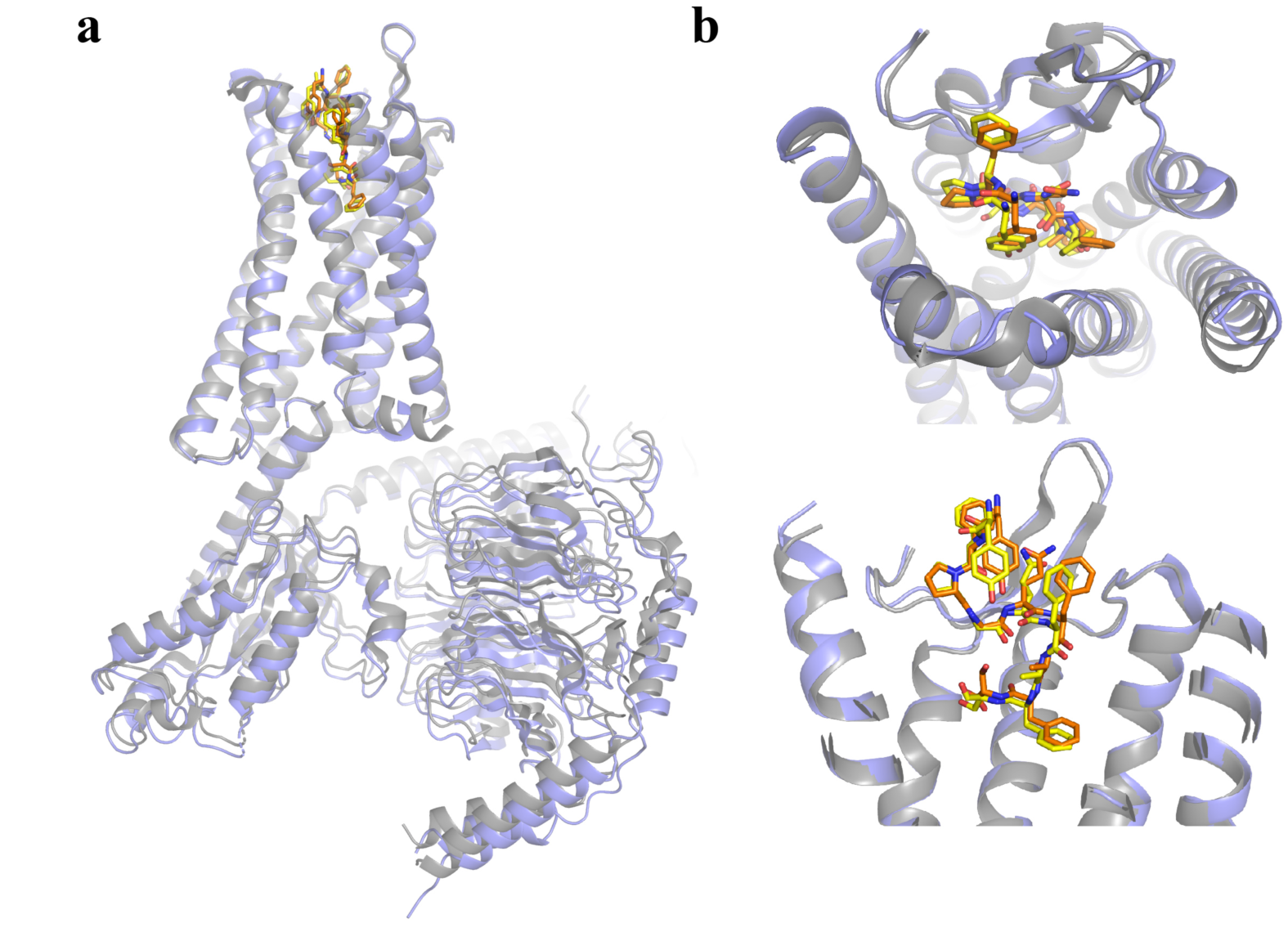

Supplement: S7 Fig — (a and b) The comparison of the overall structure and the chemerin9 binding pocket, respectively. Chemerin9 in our structure and in the published structure is colored orange and yellow, respectively. The structural alignment is based on the receptor. (TIF) [file pbio.3002188.s007.tif]

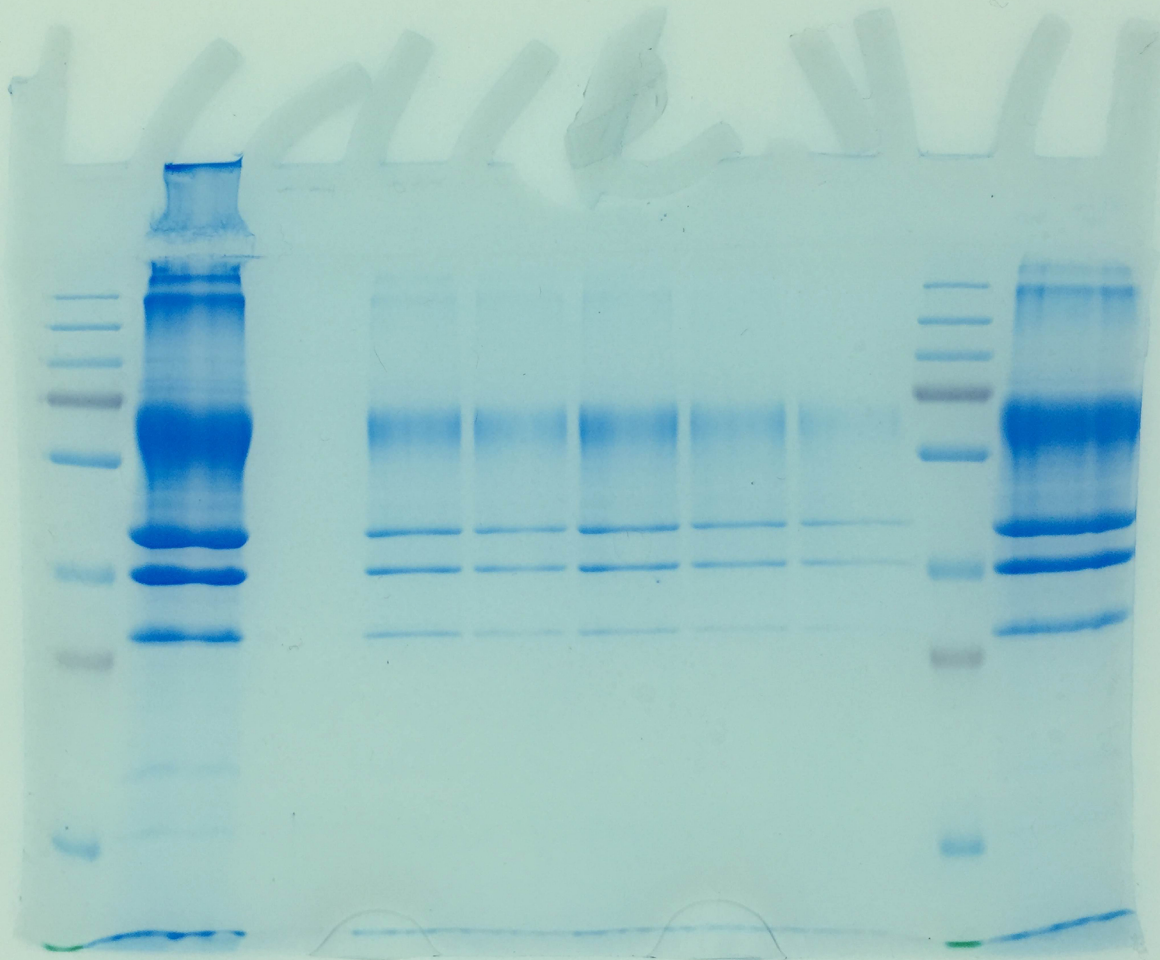

---

**Fractions of the  
sample peak**

**Concentrated  
sample**

Supplement: S1 Raw image — (PDF) [file pbio.3002188.s014.pdf]
