# Supplementary material for: Structural basis of G protein–Coupled receptor CMKLR1 activation and signaling induced by a chemerin-derived agonist
Source: PLoS Biol. 2023 Dec 6;21(12):e3002188. doi: 10.1371/journal.pbio.3002188 (PMC10699647; doi:10.1371/journal.pbio.3002188)
Supplement: Attachment — Submitted filename: ResponsesToReviewers_ChemR23_2023_2.pdf [file pbio.3002188.s015.pdf]

Manuscript ID: PBIOLOGY-D-23-01305R1

Title: **Structural basis of CMKLR1 signaling induced by chemerin9**

We thank all reviewers for their constructive comments. Please see our detailed responses to the comments below. The reviewers' comments are in **blue** font and our responses are in **black** font.

Reviewer #1 :

In the article "Structural basis of CMKLR1 signaling induced by chemerin9" Zhang et al. describes the human CMKLR1-Gi complex structure bound to chemerin9, MD simulations and functional assays together to investigate the activation mechanism. Upon receiving the manuscript, I was noted that a competing paper was already published in PNAS in March 2023. Below list some of my concerns over the model and interpretations.

Major concern

1. The overall model of CMKLR1 complex is well fit to density map, but near extracellular side there is a large amount of residues missing their density. It is particularly worrying that backbone density is also missing in some areas. This could significantly mislead readers or end users of the model. Some side chains should be trimmed to avoid confusions.

We have refined our structure further. Now we deleted regions without clear backbone density and stabbed the side chains of residues in the extracellular region with no clear density. We have revised our manuscript, figures, and tables accordingly. We hope to note that our 2.94-Å cryo-EM map is with a high quality compared to the cryo-EM maps of many other reported GPCR structures. The density of the receptor and the peptide ligand is clear to allow confident modeling of most residues with their side chains.

2. It has been reiterated in the manuscript that the pi-pi interaction contributes to the binding of chemerin9 to the receptor. It appears to me that the authors seem to be abusing this term. For example, they stated "This residue and Phe150 of chemerin9 form π-π interactions with F2947.31 (Ballesteros-Weinstein numbering) and F190ECL2 of CMKLR1, respectively". However, Phe150 is at least 12 Å away from F294. How is any interaction possible? In addition, there is merely no density to support the side chain of Phe150. If it was indeed a good pi-pi interaction between Phe150 and F190, why was it too difficult to see its density? See the screenshot below.

We apologize for the mistake. Phe150 of chemerin9 is indeed far away from F294 of CMKLR1. There is no interaction between the two residues. The density of Phe150 is weak. Nevertheless, partial density for this residue is still observed (**Fig. 1**), indicating a potential π-π interaction with $F190^{ECL2}$ of CMKLR1. It is possible that such π-π interaction is not strong enough to stabilize Phe150 in one conformation. We have revised our statement accordingly to include such information.

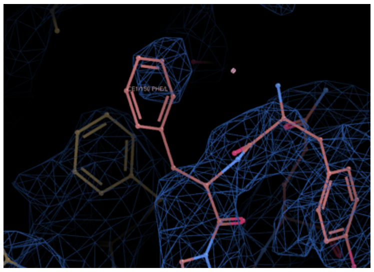

Figure 1. Partial density of Phe150 at the level of 0.13.

Another example, "At the extracellular region of CMKLR1, the first N-terminal residue of

chemerin9, Tyr149, forms a π-πinteraction with F2947.31 of CMKLR1". Although there might be a pi-pi interaction between two stated residues, it should be noted two residues at the so-called offset position are 6 Å apart. The authors need to double-check if such pi-pi interaction can be acclaimed. See the screenshot below.

The strength of pi-pi interaction is dependent on the X and Y projections of the distance between the center of the two aromatic groups, which are ~4.3Å and ~4.1Å for the Tyr149 (chemerin9) and F294 (CMKR1) pair (**Fig. 2**). According to a previous paper from Cohen et al (1) (PMID: 18260101, Figure 5), there could be a weak pi-pi interaction. We have revised our manuscript to state that "At the extracellular region of CMKLR1, the first N-terminal residue of chemerin9, Tyr149, likely engages in a weak π-π interaction with F294$^{7.31}$ of CMKLR1" and cited the paper.

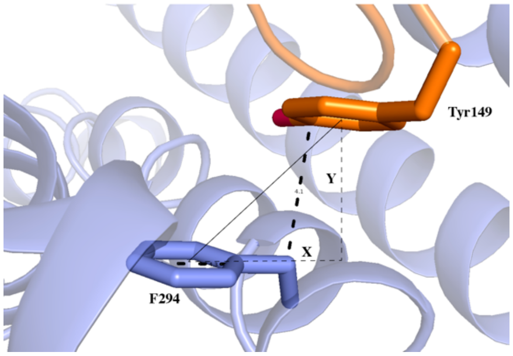

Figure 2. X and Y projections of the distance between the center of Tyr149 (chemerin9) and F294 (CMKLR1).

Also, "Phe156 of chemerin9 plays a crucial role in the interactions with the receptor, forming hydrophobic and π-π interactions with CMKLR1 residues F882.53, H952.60, M1233.36, Y2766.51, and I3067.43…". Please check if the orientation of Phe156 relative to F88 and Y276 aligns with the definition of pi-pi interaction.

Indeed, Y276 is not in an appropriate conformation to form pi-pi interactions with Phe156. We have revised our statement as "forming hydrophobic or π-π interactions with CMKLR1 residues F882.53, H952.60, M1233.36, Y2766.51, and I3067.43…"

3. The use of AlphaFold structure to represent the inactive state of CMKLR1 is unsound. First, the AlphaFold structure itself does not acclaim to be at the inactive state. Second, based on homology modeling, the source of initial models is unknown to users. Given these considerations, it is usually not used as an evidence to make comparisons between the active and inactive GPCR structures. Especially, discussing residue movements in so many details is not necessary.

We did not just use an AlphaFold-predicted structure. Instead, we used the inactive structure of CMKLR1 predicted by AlphaFold2-MultiState (2). While AlphaFold2 used machine learning methods rather than homology modeling to better predict protein structures, the results may not be entirely precise, as pointed out by the reviewer. We now include a statement as "It is to be noted that the AlphaFold-predicted inactive GPCR structures may not be entirely precise. Our proposed CMKLR1 activation mechanism, which involves conformational changes of Ser157 and Phe156 of chemerin9 and N116$^{3.29}$ and L119$^{3.32}$ of CMKLR1, only represents a plausible hypothesis." at the end of the section "An 'S-shape' binding mode of chemerin9 to induce CMKLR1 activation".

Minor concerns

1. When discussing the simulation results, the authors seem to be quite surprised that chemerin9 as a potent agonist is able to stabilize the active state of CMKLR1 in the absence of Gi. The reasons for this uncommon view are neither explained in the manuscript nor found in the reference.

Our simulations indicated that the receptor in the CMKLR1-Gi complex exhibited more conformational fluctuations without chemerin9 compared to with chemerin9. Therefore, we concluded that chemerin9 may further stabilize the active conformation of CMKLR1 beyond the stabilization caused by Gi.

Although we didn't discuss whether chemerin9 alone without Gi can stabilize the receptor in an active conformation in our manuscript, previous studies on other GPCRs including β2-adrenergic receptor (3-5) and opioid receptors (6, 7) suggested that agonists alone may not be able to stabilize active conformations of GPCRs. Instead, they could potentially enhance the likelihood of active state sampling by the receptors or reduce the energy barrier for receptor activation. For many GPCRs, agonist-binding at the extracellular region is usually loosely coupled to receptor activation at the cytoplasmic region. In the case where an agonist is bound to the receptor without its cognate G protein, the inactive conformation often remains the most thermodynamically stable for the receptor. G proteins, on the other hand, can stabilize the fully active conformations of GPCRs.

2. The authors also stated "This observation suggests that the active CMKLR1 conformation favors a close contact between the peptide agonist and the transmission switch motif." However, the two residues (Figure 6), Phe156 and W275, are at least 5.5 Å apart even when Gi is bound, which is not a close distance. Some could even say they do not contact one another.

We thank the reviewer for the suggestion. We now changed the statement to "...favors a close distance between the peptide agonist the transmission switch motif."

3. The FSC cutoff for model-to-map is 0.5 instead of 0.143.

We apologize for the mistake. We did use FSC cutoff of 0.5 for model building. We have corrected this mistake in our revised manuscript.

4. Re-examining thoroughly how well the model is fit to density is necessary for this study. For example, Glu283 of the receptor is obviously laying outside its density.

We have refined the structure further. We now deleted regions and stubbed the side chains of residues with no clear density.

5. Comparisons between current study and previously published results in PNAS should be addressed in more volumes and figures.

The two structures are highly superimposable. We have included a new **Fig. S7** to illustrate the structural alignment together with additional discussion in the last paragraph of the Discussion section.

Reviewer #2 (Dmitry Veprintsev, signs review):

The manuscript Structural basis of CMKLR1 signaling induced by chemerin9 reports the CryoEM structure of CMKLR1 aka Chem23R in complex with one of its peptide agonists. ChemR23 is implicated in inflammation and peripheral pain. In addition to its peptide agonist, it also suggested to mediate the effects of RvE1, a pro-resolving lipid. The authors showed that inducing primary human macrophages with chemerin results in a non-canonical phenotype of the macrophages, an observation consistent with its both pro-inflammation and pro-resolving roles

reported in the literature. The primary focus of the manuscript is structural analysis of the CMKLR1/Gi complex. The authors obtained a high-resolution structure, performed mutagenesis of the key residues interacting with the ligand to confirm and delaminate their role in the ligand binding or in some case also being important for activation. They also performed limited structure-activity
analysis of the chemerin9 peptide, and further investigated the roles of charged interactions by MD.

Overall, this is an excellent structural biology study, providing the first experimental structure of the important pharmacological target, and a very detailed description of the ligand-binding site. Congratulations to the authors as obtaining a structure of GPCR/G protein complex is always challenging. It will be very useful for structure-based drug design of this important pharmacological target.

Addressing a few minor points would really improve the readability of the manuscript and explain its importance to a non-expert.

1. The problem statement - what exactly the authors were aiming to achieve needs to be clearly formulated. There are several problems mentioned in the introduction 1) The apparent contradictory functional roles of CMKLR1 … (2) However, it is not clear whether RvE1 can directly bind to and activate CMKLR1 to induce Gi signaling and (3) However, the lack of synthetic ligands of CMKLR1. Which one of the problems? The solution is the structure but what was the problem?

We thank the reviewer for the suggestion. All of them are significant focal points for the study of CMKLR1. Our study is mainly focused on peptide agonists of CMKLR1. We sought to determine whether chemerin9 induces a pro-inflammatory or pro-resolving phenotype of macrophages and how this peptide agonist interacts with and activates CMKLR1. In the Discussion section, we discussed insights from our structure into the potential binding of RvE1. We also revised our Introduction section by including a statement at the end as "The structural information is anticipated to facilitate the development of synthetic small molecule agonists mimicking the action of chemerin9 for CMKLR1."

2. There are 67 ligands reported for this receptor in gpcrdb.org as of today, although this may not have been the case when the project was initiated. Some of the argumentation may need to be revised to reflect this. Depending on the problem statement the authors would choose, this may provide an opportunity to discuss drug design in structural context.

The argumentation in our manuscript regarding CMKLR1 ligands stands accurate. Most of reported ligands for CMKLR1 are chemerin and its derived peptides, with many developed from the lab of Dr. Annette Beck-Sickinger, a co-corresponding author of our paper. While some lipid ligands have been proposed for CMKLR1 such as RvE1, it remains controversial as if those are genuine endogenous CMKLR1 ligands. At the moment, no synthetic small molecule agonists of CMKLR1 have been reported. We firmly believe that the elucidated structure can serve as a framework for the development of small molecule agonists for CMKLR1. We have discussed this point in the Discussion section.

3. A potential binding site for RvE1 suggested on Fig 3b. Again, depending on the problem

statement this could be given a more prominent position in the manuscript.
At least a part of RvE1's pro-resolving function has been linked to CMKLR1 signaling. However, the direct binding of RvE1 to CMKLR1 remains a topic of ongoing debate. Given this uncertainty, we didn't extensively discuss the specific binding mechanism of RvE1 to CMKLR1. Instead, we simply suggested that the region above ICL2 for the binding of lipids might potentially accommodate RvE1. However, an important point is that this site could be utilized to develop allosteric modulators for CMKLR1. A similar site right above ICL2 of C5aR, a close phylogenetic neighbor of CMKLR1, has been targeted by many allosteric modulators. We have included this information in the Discussion section.

4. Discussion: it is very concise but reads more like an introduction. It really needs to discuss how the new results addressed the problem statement stated in the beginning of the manuscript. Once the problem statement is clarified, it would be easier to focus the discussion. The discussion would also benefit from a short 3-4 lines conclusion summarising the key fundings.

We thank the reviewer for the suggestion. We have revised our Discussion section accordingly to include a short 3-4 lines summary in the first paragraph. In the second paragraph, we mainly discussed how our structure informs drug development on CMKLR1.

5. Technical comment: The details of staining of the receptor in microscopy (Fig. S5) is not described anywhere. One can guess it is likely to be an anti-FLAG fluorescent Ab staining but this needs to be included in the methods.

We used CMKLR1-eYFP constructs. The expression of receptor was measured by eYFP autofluorescence. We have included the information in the Methods and the Supplementary Data.

Reviewer #3 :

The manuscript by Zhang et al describes the cryo-EM structure of CMKLR with chimerin9 and a Gi heterotrimeric G protein. This is second structure (and paper) describing this complex, nevertheless it is a very well performed study that adds to the understanding of how chimerin9 binds to and activates CMKLR.
The foundation for this study is the cryo-EM map of the complex that was determined to 2.9Å resolution. This map has excellent density for all parts of the ligand and coupled with well-performed modelling creates a solid foundation for the extensive mutagenesis and ligand structure-function studies authors undertook. With the exception of some minor points listed below, I fully recommend this manuscript for a publication in PLOS Biology.

Minor comments (these are not essential for understanding the manuscript but would improve it quality):
1. Cholesterol modelling in the map is a bit too ambitious- this density is too noisy/low contoured/uncertain to even assign a particular lipid

As suggested by the reviewer, we have deleted one cholesterol molecule due to the noisy density and revised **Fig. S3** accordingly.

2. Confusing labels on Fig. S6 as some panels have G proteins and some do not.

We have revised Fig. S6a and the figure legend to make it clearer. The left and right panel represent MD simulations of two systems, the CMKLR1-G_i complex with chemerin9 (left) and without chemerin9 (right). In each panel, RMSDs of receptor alone and of the CMKLR1-G_i complex are shown in the upper and lower graphs, respectively.

3. Fig. 6b- A bit weird that the cryo-EM structure did not capture the captured the closest distance of W273 and chimerin9 - a discussion sentence on that in the text would be very helpful.

It is possible that the closet distance between W273 and chemerin9 may not be the most energetically favorable state but only represent a local minimum energy state, which was only captured by our MD simulations. We have included additional discussion in the main text.

4. Could the explanation for "the CMKLR1-Gi complex, the Gi heterotrimer remained stably bound to the receptor, regardless of the presence of chemerin9 (Fig. S6a)." be that the simulation is too short? Authors only run the 100ns simulation, while it is my understanding that to assess G protein binding at G proteins generally longer time frames are required (1uM). Perhaps and alternative explanation can also be added/considered.

We have performed simulations for five distinct systems with three runs of 100-ns molecular dynamics simulations for each system. We chose to conduct triplicate runs for each system under various conditions (total of 3 x 5 runs) instead of a single simulation run lasting microseconds because we believe the former approach provides statistically reproducible results, with a more accurate sampling of the vicinity of the starting conformers. On the other hand, we agree with the reviewer that the duration of these runs might not be sufficient to capture conformational transitions of the Gi heterotrimer. We have revised our statement in the main text to include the information of 100-ns simulation time frame for our MD simulations and acknowledged its limitation as "We note that such a time frame may not be long enough to capture the potential conformational transitions of Gi heterodimer during activation and dissociation.".

References

1. M. Cohen, D. Reichmann, H. Neuvirth, G. Schreiber, Similar chemistry, but different bond preferences in inter versus intra-protein interactions. *Proteins* **72**, 741-753 (2008).
2. G. Pandy-Szekeres *et al.*, GPCRdb in 2023: state-specific structure models using AlphaFold2 and new ligand resources. *Nucleic Acids Res* **51**, D395-D402 (2023).
3. D. M. Rosenbaum *et al.*, Structure and function of an irreversible agonist-beta(2) adrenoceptor complex. *Nature* **469**, 236-240 (2011).
4. R. Nygaard *et al.*, The dynamic process of beta(2)-adrenergic receptor activation. *Cell* **152**, 532-542 (2013).
5. A. Manglik *et al.*, Structural Insights into the Dynamic Process of beta2-Adrenergic Receptor Signaling. *Cell* **161**, 1101-1111 (2015).
6. W. Huang *et al.*, Structural insights into micro-opioid receptor activation. *Nature* **524**, 315-321 (2015).
7. R. Sounier *et al.*, Propagation of conformational changes during mu-opioid receptor activation. *Nature* **524**, 375-378 (2015).
