# Supplementary material for: Structural basis of G protein–Coupled receptor CMKLR1 activation and signaling induced by a chemerin-derived agonist
Source: PLoS Biol. 2023 Dec 6;21(12):e3002188. doi: 10.1371/journal.pbio.3002188 (PMC10699647; doi:10.1371/journal.pbio.3002188)
Supplement: Attachment — Submitted filename: ResponsesToReviewers_ChemR23_2023_4.pdf [file pbio.3002188.s016.pdf]

Manuscript ID: PBIOLOGY-D-23-01305R2 - [EMID:de980db009186427]

Title: **Structural basis of G-protein coupled receptor CMKLR1 activation and signaling induced by a chemerin-derived agonist**

We thank all reviewers for their constructive comments. Please see our detailed responses to the comments below. The reviewers' comments are in **blue** font and our responses are in **black** font.

Reviewer #2 :

Reviewer #2 (Dmitry Veprintsev, signs review): The revised version of the paper is more focused. It reads much better, and the authors addressed most of my comments. One minor point that could still be improved (but it does not really affect the conclusions of the paper) is that there were a few small molecule antagonists reported in the literature (and GPCRDB database under the alternative receptor name CML1). These molecules may need to be mentioned for factual correctness in the introduction and corresponding section of the discussion, with the corresponding adjustment of the wording.

https://nam12.safelinks.protection.outlook.com/?url=https%3A%2F%2Fpubmed.ncbi.nlm.nih.gov%2F32773087%2F&data=05%7C01%7Cchengzh%40pitt.edu%7Cfef8a43a19544f8b909808dbcee935d0%7C9ef9f489e0a04eeb87cc3a526112fd0d%7C1%7C0%7C638331273270403226%7CUnknown%7CTWFpbGZsb3d8eyJWIjoiMC4wLjAwMDAiLCJQIjoiV2luMzIiLCJBTiI6Ik1haWwiLCJXVCI6Mn0%3D%7C3000%7C%7C%7C&sdata=hhy4crdZoIJpe8zjVzsBTmy%2BMzWzXW5P%2FQT4JX2QhdE%3D&reserved=0
https://nam12.safelinks.protection.outlook.com/?url=https%3A%2F%2Fpubmed.ncbi.nlm.nih.gov%2F31521459%2F&data=05%7C01%7Cchengzh%40pitt.edu%7Cfef8a43a19544f8b909808dbcee935d0%7C9ef9f489e0a04eeb87cc3a526112fd0d%7C1%7C0%7C638331273270403226%7CUnknown%7CTWFpbGZsb3d8eyJWIjoiMC4wLjAwMDAiLCJQIjoiV2luMzIiLCJBTiI6Ik1haWwiLCJXVCI6Mn0%3D%7C3000%7C%7C%7C&sdata=xs%2BkcfvO3AIRj70a8OAFYUzykBiWF4Ucus5VdmDYL9s%3D&reserved=0

We have revised our manuscript accordingly and cited those studies.

Reviewer #3 :

Reviewer #3: All of my comments have been addressed. Biology.
